# Clinical Subtype Stratification Reveals Gut Microbial Alterations, Candidate Markers, and Co-Occurrence Network Remodeling in Coronary Artery Disease

**DOI:** 10.3390/microorganisms14092117

**Published:** 2026-09-21

**Authors:** Peiqi Yan, Chen Tian, Lei Zhu, Zhigang Zhang

**Affiliations:** 1 State Key Laboratory for Conservation and Utilization of Bio-Resources in Yunnan, School of Life Sciences, Yunnan University, Kunming 650091, China; page_yan@126.com (P.Y.); tcsecret@163.com (C.T.); 2 Cancer Research Institute, Yunnan Cancer Hospital, The Third Affiliated Hospital of Kunming Medical University, Peking University Cancer Hospital Yunnan, Kunming 650106, China

**Keywords:** coronary artery disease, gut microbiota, biomarkers

## Abstract

Although gut microbiota dysbiosis is associated with coronary artery disease (CAD), its dynamic alterations across distinct clinical stages remain largely unexplored. This study aimed to identify stage-specific microbial biomarkers across the CAD spectrum. We analyzed 16S rRNA sequencing data from 306 subjects, including non-CAD controls and patients with mild coronary stenosis (MCS), stable angina (SA), unstable angina (UA), and acute myocardial infarction (AMI). Diversity analyses, machine learning, and SPIEC-EASI network modeling were applied to evaluate microbial community shifts and identify core diagnostic markers. Significant structural remodeling of the gut microbiota was observed, particularly during the AMI stage. *Veillonella*, *Intestinimonas*, and *Streptococcus* were identified as the core biomarkers for the MCS, SA, and UA/AMI stages, respectively. From the MCS to the UA stage, ecological network connectivity continuously declined, highlighting critical barrier vulnerability, before transitioning into an antagonism-dominated network in the AMI stage. Machine learning models effectively discriminated among the various disease stages, and key microbial markers remained statistically robust after adjusting for major clinical confounders. The gut microbiota exhibits dynamic, stage-specific dysbiosis throughout CAD progression, providing promising non-invasive biomarkers for precise disease staging and diagnosis.

## 1. Introduction

Cardiovascular disease (CVD) is currently the leading non-communicable disease (NCD) responsible for global morbidity and mortality [1,2]. Coronary Artery Disease (CAD) has the highest incidence and mortality rates among all cardiovascular diseases globally [3] and has been the leading single cause of death worldwide for many years [4]. The pathological basis of CAD is coronary atherosclerosis [5]. In addition, risk factors such as smoking, hypertension, diabetes mellitus, abdominal obesity, poor diet, physical inactivity, and psychosocial stress all contribute to the pathogenesis of CAD [6]. CAD progression is a complex process co-regulated by systemic inflammatory status, lipid metabolism, hemodynamics, and the biological characteristics of local plaques [7,8]. In modern clinical practice, based on the compensatory state of coronary hemodynamics and the biophysical stability of the plaque itself, the clinical course of CAD is strictly classified into two major categories: chronic coronary syndromes (CCSs) and acute coronary syndromes (ACSs) [9]. These phenotypes are relatively independent but can undergo non-linear, abrupt transitions driven by specific pathological conditions [10,11].

Recently, an increasing number of studies have reported the association between gut microbiota and the development of CAD [12,13,14]. The gut microbiota influences CAD progression primarily by regulating cholesterol metabolism and bile acid synthesis. In healthy populations, specific intestinal probiotics (such as *Lactobacillus* and *Bifidobacterium*) can reduce the reabsorption efficiency of bile acids in the terminal ileum, promoting the excretion of large amounts of cholesterol derivatives through feces [15,16]. The generated short-chain fatty acids (SCFAs) can also enter the liver via the portal vein to further inhibit intrahepatic cholesterol synthesis pathways, thereby maintaining lower blood cholesterol levels in the host [17,18]. However, under pathological conditions, the abnormal activation of specific metabolic pathways mediated by the gut microbiota or the accumulation of specific metabolites can also exert adverse effects on host health [19,20]. In particular, trimethylamine N-oxide (TMAO), a gut microbiota-derived metabolite, severely inhibits the reverse cholesterol transport network of macrophages [21] and directly accelerates lipid engulfment and deposition in the arterial wall [22], acting as a major risk factor for the progression of various CVDs [23]. Furthermore, the gut microbiota can promote the initiation and progression of CAD by modulating the host’s systemic immune homeostasis and local inflammation [24,25,26]. However, differences in the gut microbiota across various clinical subtypes at different stages of CAD remain scarcely reported. While previous pioneering studies have robustly documented the overarching association between gut microbiota dysbiosis and CAD [14], these investigations frequently treat CAD as a single binary condition. Our study extends this prior work by characterizing the dynamic, stage-specific alterations and network remodeling across distinct clinical phases.

Biomarkers play an indispensable and central role in modern cardiovascular clinical practice, greatly advancing the early detection and precise prognostic stratification of the disease [27]. High-sensitivity cardiac troponins (hs-cTnI and hs-cTnT) have become the recommended modalities for the diagnosis and risk stratification of CAD in clinical settings [28]. Apolipoprotein B (ApoB), lipoprotein(a) [Lp(a)], and specific heart-enriched microRNAs within cardiomyocytes have also been reported to possess potential as novel biomarkers [29,30]. Nevertheless, their ability to discriminate among distinct stages of CAD has rarely been reported.

To identify gut microbiota associated with the development of CAD, we re-analyzed 16S rRNA sequencing data from fecal samples previously collected from non-CAD controls and CAD patients, who were classified based on the degree of hemodynamic obstruction of the coronary lumen, functional manifestations of myocardial ischemia, and the biological risk of plaque rupture. The clinical subtypes of CAD included mild coronary stenosis (MCS), stable angina (SA), unstable angina (UA), and acute myocardial infarction (AMI). By analyzing the characteristics and network structural alterations of the gut microbiota in patients with different clinical subtypes to screen for microbial biomarkers, our results provide novel insights into the potential role of the gut microbiota in the development, diagnosis, and treatment of CAD.

## 2. Materials and Methods

### 2.1. Data Collection

To investigate the global microbial signatures across different stages of CAD, we analyzed the 16S rRNA sequencing data from our previous study conducted with Yan’an Affiliated Hospital of Kunming Medical University [31]. The BioProject ID in the NCBI Sequence Read Archive (SRA) database is PRJNA779598. This dataset contains paired-end sequencing data of the V3-V4 region of fecal samples from 65 non-CAD subjects and 241 CAD patients at different stages from the Kunming area, diagnosed according to the 2020 European Society of Cardiology (ESC) guidelines [32].

To maintain consistency with the original cohort’s baseline and avoid introducing computational batch effects, we utilized the previously validated operational taxonomic unit (OTU) abundance table (Table S2) [31] rather than reprocessing the raw FASTQ files.

Briefly, the V3–V4 region of the 16S rRNA gene was sequenced using paired-end technology on the Illumina MiSeq platform. The raw sequencing data were imported into the QIIME 2 pipeline [33] for initial preprocessing, which included the removal of adapters and primers, paired-end read merging, and low-quality sequence filtering. Subsequently, the DADA2 plugin [34] was employed for data denoising—encompassing quality score filtering, dereplication, and chimera removal—to generate the Amplicon Sequence Variant (ASV) abundance table. To eliminate potential biases caused by varying sequencing depths across samples, the ASV table was randomly rarefied to an even depth of 10,000 sequences per sample. For taxonomic annotation, representative ASV sequences were classified using the RDP naive Bayes classifier algorithm [35] against the Ribosomal Database Project (RDP) 16S rRNA training set (v16) database [36].

The clinical groups included in the analysis were Non-CAD Control, AMI, UA, SA, and MCS. The classification is as follows:

(i). Mild coronary stenosis (MCS) was defined as a coronary lumen stenosis of <50% as shown by coronary angiography;
(ii). Stable angina (SA) was defined as a condition causing exercise- and stress-related chest symptoms due to a stenosis of more than 50% in the left main coronary artery or more than 70% in one or more major coronary arteries;
(iii). Unstable angina (UA) was defined as myocardial ischemia occurring at rest or with mild exercise, without non-ST-segment (S wave and T wave) changes related to acute myocardial cell injury/necrosis on the electrocardiogram;
(iv). Acute myocardial infarction (AMI) was defined by acute chest pain and myocardial cell necrosis, encompassing both ST-segment elevation myocardial infarction (STEMI) and non-ST-segment elevation myocardial infarction (NSTEMI);
(v). Participants diagnosed with no CAD were recruited as the non-CAD control group.

The clinical information of the samples mainly consisted of age, body mass index (BMI), fibrinogen (FBG), glycated hemoglobin (HbA1c), total cholesterol (CHOL), triglycerides (TG), creatine kinase (CK), creatine kinase isoenzyme (CK-MB), lactate dehydrogenase (LDH), and cardiac troponin I (cTnI).

Based on the clinical labels provided in Table S1 [31], a total of 306 subjects were included, comprising 65 non-CAD controls, 36 with MCS, 48 with SA, 91 with UA, and 66 with AMI.

### 2.2. Processing of Clinical Data and Microbial Abundance Profiles

The clinical metadata and the OTU abundance table were merged by Subject ID. Only samples with valid clinical group assignments present in both datasets were retained. Taxonomic strings were parsed into kingdom, phylum, class, order, family, genus, and species levels, with OTU counts subsequently aggregated at the genus level. Relative abundances were calculated by dividing the count of a specific taxon within a sample by the total count of that sample. For the Bray–Curtis dissimilarity analysis, OTUs were required to have a relative abundance of at least 0.1% in at least one sample and be detected in a minimum of two samples; this filtering step retained 1721 out of the original 3627 OTUs. For the genus-level differential abundance analysis and machine learning models, bacterial genera with a detection rate of at least 5% were separately retained for each pairwise comparison between a CAD subtype and the control group.

### 2.3. Comparison of Baseline Clinical Characteristics

Continuous variables were presented as medians and interquartile ranges (IQRs), and comparisons among the five groups were performed using the Kruskal–Wallis test. Categorical variables were expressed as counts and percentages and were compared using Pearson’s chi-square test. The *p*-values of all baseline variables were adjusted for the false discovery rate (FDR) using the Benjamini–Hochberg method [37]. For continuous variables, an available-case analysis was applied, and the number of non-missing samples in each group was reported.

### 2.4. Alpha Diversity, Beta Diversity, and Community Composition

Based on the normalized OTU counts, the Observed OTUs, Chao1, Shannon, Simpson, and Pielou’s indices were calculated. Overall differences among the five groups were evaluated using the Kruskal–Wallis test, followed by post hoc comparisons between each disease subtype and the control group using two-sided Mann–Whitney U tests. The *p*-values from all 25 alpha diversity tests were uniformly adjusted using the Benjamini–Hochberg method. Additionally, a sensitivity analysis was performed by combining the four CAD subtypes to compare against the control group.

Bray–Curtis dissimilarities were calculated based on the filtered OTU relative abundances and subjected to classical Principal Coordinate Analysis (PCoA) for dimensionality reduction. Differences in community structure among the five groups and between the combined binary classification (CAD vs. Control) were assessed using permutational multivariate analysis of variance (PERMANOVA) [38] with 999 permutations (random seed = 42). The pseudo-F statistic, *R*^2^, and permutational *p*-values were reported. Dispersions were compared utilizing the Euclidean distances from individual samples to their respective group centroids within PCoA coordinates featuring positive eigenvalues, and inter-group distances were assessed via a one-way analysis of variance (ANOVA). For taxonomic composition plots at the phylum and genus levels, the top 10 and 15 taxa with the highest mean abundances were retained, respectively, while the remaining taxa were aggregated into “Others”.

### 2.5. Differential Genera, Marker Scores, and Weight Sensitivity

In the binary classification framework of each subtype versus the control, the relative abundance matrix at the genus level was used as input features, and a random forest model [39] was employed to evaluate the discriminatory ability of microbial features. The model parameters were set as follows: n_estimators = 1200, class_weight = balanced_subsample, max_features = sqrt, and min_samples_leaf = 2. The model performance was evaluated through RepeatedStratifiedKFold cross-validation, with 5-fold cross-validation repeated 5 times and a random seed of 42. The model outputted the average AUC and its standard deviation, and feature importance was extracted for the comprehensive scoring of candidate markers.

Given the compositional nature of 16S relative abundance data, the genus-level counts were first subjected to a centered log-ratio (CLR) transformation [40] after adding a pseudocount of 0.5. Differences between each disease subtype and the control group were evaluated using two-sided Mann–Whitney U tests, and the false discovery rate (FDR) was calculated within each subtype using the Benjamini–Hochberg method. Concurrently, the mean CLR difference (disease group minus Control), alongside the detection rates (prevalences) and prevalence differences between the two groups, were computed. Random Forest models were constructed using genus-level relative abundances as inputs. The internal model hyperparameters were set to 600 trees, ‘balanced_subsample’ class weights, square root feature sampling, and a minimum of 2 samples per leaf node. The models were evaluated using 5-fold stratified cross-validation repeated 3 times, with a random seed set to 42. Additionally, a separate model consisting of 800 trees was fitted on the complete dataset comprising the specific subtype and control samples to extract feature importances. To enhance the interpretability of candidate markers, this study introduced a comprehensive scoring strategy:

$$\begin{array}{ll} Marker\;score= & 0.35\times Significance\;score+RF\;importance\;score\\ & +\;0.20\times Effect\;size\;score+0.15\times Prevalence\;score \end{array}$$

Within each subtype, the statistical significance, Random Forest importance, absolute CLR effect size, and absolute prevalence difference were Min-Max normalized to a scale of 0 to 1. The Marker Score was then calculated as a weighted sum using initial weights of 0.35, 0.30, 0.20, and 0.15, respectively.

Based on these rankings, the top 15 genera in each subtype were extracted for subsequent candidate analysis, with the top 8 highest-scoring features selected for presentation in the main text. Weight sensitivity analyses included equal weighting of the four components and a leave-one-out approach (systematically omitting one component at a time). During the leave-one-out process, the remaining original weights were re-normalized to sum to 1, followed by an evaluation of intra-group ranking comparisons. The final score solely indicates the priority of the candidates.

### 2.6. Clinical Spectrum Ordering and Core Genera

The non-CAD Control, MCS, SA, UA, and AMI groups were sequentially encoded from 0 to 4 according to a predefined clinical spectrum order. Spearman correlation coefficients were calculated between the CLR-transformed abundance of each genus and this ordinal ranking, followed by multiple testing correction across all genera using the Benjamini–Hochberg method. It should be noted that this analysis reflects cross-sectional associations across the clinical spectrum and is not intended to imply longitudinal disease progression within individual subjects. Genera demonstrating a prevalence of 50% across all five groups were designated as cross-group core genera.

### 2.7. SPIEC-EASI Networks and Topological Metrics

Across all samples, genera with a prevalence of 15% and a mean relative abundance of 0.05% were initially filtered. Subsequently, the top 40 genera with the highest mean abundance were selected as the common candidate nodes to ensure consistency across the five groups. Within each group, the conditional dependence network was independently estimated utilizing the Meinshausen–Buhlmann (MB) method [41] implemented in the SPIEC-EASI framework [42]. The regularization path was constructed with 30 λ values and a minimum λ ratio of 0.01. Model selection was performed using the Stability Approach to Regularization Selection (StARS) [43] with 50 subsampling iterations and an instability threshold set to 0.10. Group-specific random seeds were assigned incrementally, starting from 20260714. Calculated topological metrics for the networks included the number of retained nodes, total edges, positive and negative edges, average degree, network density, global clustering coefficient, Louvain modularity, and the number of connected components. It should be emphasized that the network edges represent statistically inferred conditional dependencies rather than direct biological microbial interactions. Furthermore, inter-group comparisons of the topological metrics were strictly descriptive.

### 2.8. Comparison of Machine Learning Models

Binary classification models were individually constructed to compare each disease subtype (MCS, SA, UA, and AMI) against the Control group. Genus-level relative abundances with a prevalence of 5% were utilized as input features. The performance of four machine learning algorithms was compared: Random Forest [39], L1-regularized Logistic Regression [44], linear Support Vector Machine (SVM) [45], and radial basis function (RBF) kernel SVM [46]. For non-Random Forest models, feature standardization was integrated into the training pipeline. Class imbalances were addressed by applying ‘balanced’ or ‘balanced_subsample’ class weights. All models were rigorously evaluated using a 5-fold stratified cross-validation repeated 3 times, and the mean and standard deviation of the area under the receiver operating characteristic curve (AUC) across the 15 validation folds were reported.

### 2.9. Correlations Between Markers and Clinical Indices, and Covariate Adjustment

Spearman correlation analyses were performed between the CLR-transformed abundances of candidate genera and clinical indices, including age, BMI, FBG, HbA1c, CHOL, TG, CK, CK-MB, LDH, and cTnI. Combinations with fewer than 20 valid samples, or those exhibiting zero variance in either the genus abundance or the clinical parameter, were excluded from statistical testing. The *p*-values for all remaining comparisons were uniformly adjusted using the Benjamini–Hochberg method.

For the top 15 candidate genera identified within each subtype, ordinary least squares (OLS) regression models were constructed to compare the specific subtype against the Control group. In these models, the CLR abundance was designated as the dependent variable (outcome), with disease status serving as the primary independent variable. The models were adjusted for potential confounders, including age, BMI, male sex, hypertension, type 2 diabetes mellitus, and hypertriglyceridemia. A complete-case analysis approach was employed, and the adjusted CLR regression coefficients for disease status, along with their corresponding *p*-values and sample sizes, were reported. Finally, the *p*-values derived from all 60 models were uniformly subjected to false discovery rate (FDR) correction.

### 2.10. Software and Statistical Standards

All secondary analyses were performed in a 64-bit Windows 11 environment. Data cleaning, matching of samples with clinical metadata, taxonomic aggregation, and result table curation were executed in Python 3.12.10, utilizing pandas 3.0.3 [47] and NumPy 2.4.6 [48]. The reading and writing of Excel files were handled by openpyxl 3.1.5 [49]. Distance calculations, non-parametric tests, correlation analyses, and analysis of variance (ANOVA) were performed using SciPy 1.17.1 [50]. Machine learning implementations—including Random Forest, L1-regularized Logistic Regression, and Support Vector Machines (SVM) with linear and radial basis function (RBF) kernels—along with repeated stratified cross-validation and AUC calculations, were conducted using scikit-learn 1.8.0 [51]. Co-occurrence network estimations were conducted in R 4.6.0 using SpiecEasi 1.99.0 [52], and network topological metrics were computed using igraph 2.3.2 [53]. Data organization for fixed-coordinate networks was managed with NetworkX 3.6.1 [54]. Main and supplementary figures were generated utilizing Matplotlib 3.10.9 [55] and Seaborn 0.13.2 [56]. Unless otherwise specified, all statistical tests were two-sided. Multiple comparisons were adjusted using the Benjamini–Hochberg method, with a false discovery rate (FDR) < 0.05 established as the threshold for statistical significance following correction.

## 3. Results

### 3.1. Sample Composition and Clinical Baseline Characteristics

**Figure 1:**
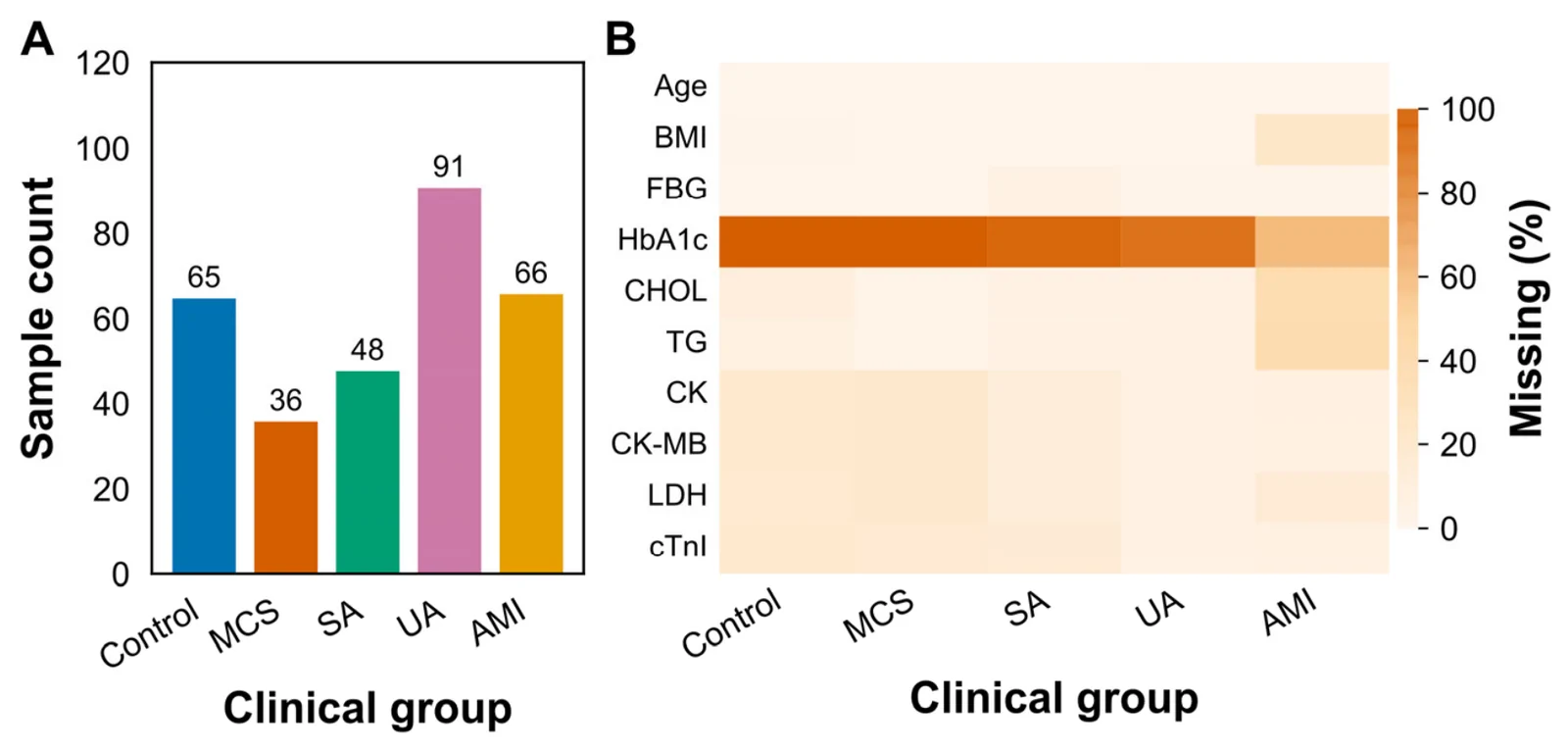
Sample composition and missingness of clinical variables across the five clinical groups. (**A**) Number of samples included in each group. (**B**) Missing proportions of each clinical variable across the five groups (%, 0–100%); darker colors indicate higher missing proportions, with exact percentage values annotated in each cell.

The 306 samples included in the analysis were categorized into five groups: Non-CAD Control, MCS, SA, UA, and AMI, with sample sizes of 65, 36, 48, 91, and 66, respectively (Figure 1A). Overall inter-group comparisons for age, CK, CK-MB, LDH, and cTnI all reached statistical significance following FDR correction. The detailed baseline distributions and statistical results are summarized in Table 1.

Compared to the other four groups, the AMI group exhibited pronounced pathological changes. Myocardial injury biomarkers, including cTnI, CK, CK-MB, and LDH [57], demonstrated highly significant pathological elevations in the AMI group (*p* < 0.001), whereas they remained at extremely low or baseline levels in the control group and other CAD subgroups (Table 1). This finding is consistent with the Fourth Universal Definition of Myocardial Infarction [28]. The significant elevation of biomarkers such as cTnI confirmed that the group assignment based on the key clinical phenotype of acute cardiomyocyte necrosis in our cohort was accurate and reliable. Regarding demographic characteristics, the AMI group presented an exceptionally high proportion of males (92.4%, Table 1), which aligns with global cardiovascular epidemiological data, reflecting the estrogen-mediated protection in premenopausal women and the higher early susceptibility of men to the pathogenesis of ACS [58,59].

In terms of comorbidities, hypertension, acting as a core risk factor driving vascular endothelial dysfunction [58], accounted for highly substantial proportions in the MCS (80.6%) and UA (65.9%) groups (Table 1). Conversely, cardiac neurosis was exclusively detected in the control group (26.2%, Table 1). This aligns with the clinical exclusion logic in cardiology: a large number of patients presenting with chest pain but ultimately showing negative coronary angiography results are frequently attributed to non-cardiogenic anxiety or neurosis [60]. Furthermore, coronary myocardial bridging was relatively common in the control and MCS groups but extremely rare in the severe ischemic UA and AMI groups (Table 1), suggesting that congenital anatomical variations in vascular morphology are not the primary driving factors in such acute plaque rupture events [61].

The missing rates of clinical indices are illustrated in Figure 1B. With the exception of a relatively high missing rate for HbA1c in certain groups, the missing rates for the other presented clinical parameters were low. The sample sizes of the five groups and the missing patterns of clinical variables, together with Table 1, defined the sample composition for subsequent stratified comparisons. Complete sequencing depth and OTU filtering information are detailed in Figure S1 and Table S1. The medians and means of sequencing depth across all five clinical groups were highly comparable (Figure S1A). From the raw OTU set obtained through initial clustering or denoising, a total of 1906 OTUs were filtered out as low-quality or statistically insignificant features, ultimately retaining 1721 high-quality OTUs for downstream analyses (Figure S1B).

### 3.2. Overview of Diversity, Structure, and Composition

We analyzed the overall alpha and beta diversities between the Control and CAD groups. The baseline architectural framework (Figure S2A–C) and taxonomic compositional proportions (Figure S2D) of the gut microbiota in CAD patients did not exhibit any significant macroscopic deviations from those in the non-CAD population. Complete diversity metrics and overall test results are presented in Table S2. This highlights the necessity of further stratifying CAD into distinct disease stages (MCS, SA, UA, and AMI) for multiple-group comparisons.

**Figure 2:**
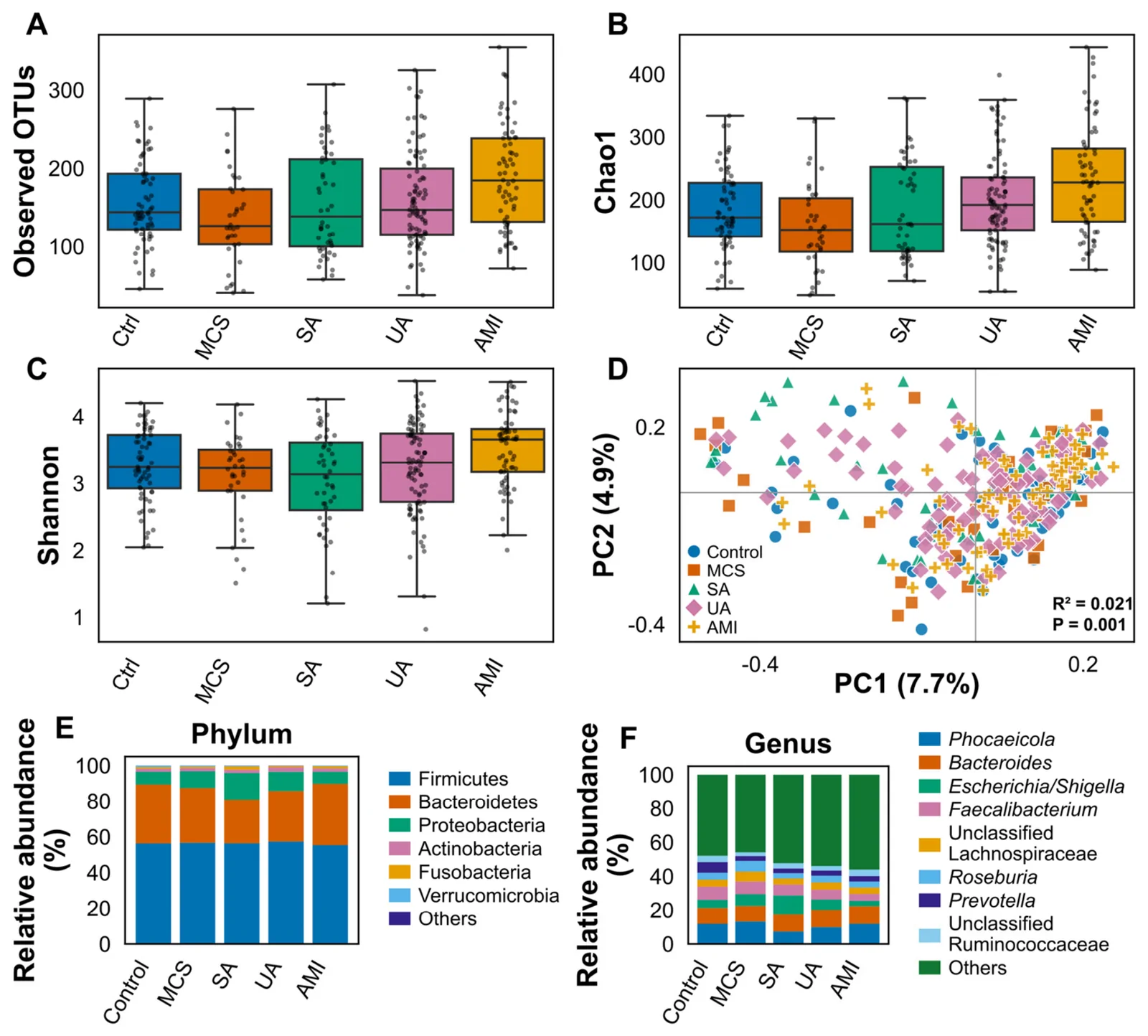
Alpha diversity, beta diversity, and community composition across the five clinical groups. (**A**–**C**) Observed OTUs, Chao1, and Shannon indices; (**D**) Bray–Curtis PCoA based on filtered OTU relative abundances; (**E**,**F**) group-mean relative abundances of the top 10 phyla and 15 genera with the highest average abundances. Taxa are ordered in descending order of mean abundance across groups, and the remaining taxa are merged into “Others”.

In the five-group comparisons across different disease stages, the overall differences in Observed OTUs, Chao1, and Shannon indices remained statistically significant following FDR correction (Figure 2A–C). Notably, the differences in Observed OTUs and Chao1 indices were not uniformly distributed across the various CAD stages; rather, they were primarily concentrated between the acute myocardial infarction (AMI) group and the Non-CAD Control group (post hoc FDR = 0.029 and 0.019, respectively; Figure 2A,B). The complete test results for Simpson and Pielou’s indices are detailed in Table S2. This implies that during the stages of angina (SA, UA) or mild coronary stenosis (MCS), the richness of the gut microbiota might be maintaining a baseline equilibrium via certain compensatory mechanisms; however, once the disease breaches the critical threshold and progresses to AMI, the community richness undergoes an abrupt shift [20,62]. From a pathophysiological perspective, the onset of AMI is accompanied by drastic hemodynamic alterations, extreme sympathetic nervous system hyperactivation [63], and a systemic inflammatory storm [64]. Such extreme acute physiological stress can directly lead to intestinal microcirculatory hypoperfusion, intestinal mucosal ischemia and hypoxia, as well as intestinal barrier dysfunction [65,66]. This consequently triggers the abnormal proliferation of specific tolerant strains or the rapid depletion of sensitive commensal bacteria [67], ultimately manifesting as significant aberrations in community richness metrics within the sequencing data.

Beta diversity analysis further corroborated this stage-specific remodeling of the microbiota at a macroscopic structural level. PERMANOVA based on Bray–Curtis distances confirmed highly significant structural differences in the microbial community compositions among the five clinical groups (*p* = 0.001), with the disease stage accounting for approximately 2.1% of the overall community variation (R^2^ = 0.021). In the context of highly heterogeneous gut microbiome studies, this effect size is sufficient to demonstrate the substantial impact of pathological states on microbiota structure [67]. Furthermore, the non-significant result of the PERMDISP test (*p* = 0.137) statistically precluded the possibility of false positives caused by unequal intra-group variances, thereby confirming that the observed differences genuinely originate from objective shifts in community centroids across the distinct disease stages.

Regarding the group-average relative abundance profiles at the phylum and genus levels, differences in microbial composition could be visually observed by comparing the top 10 phyla (Figure 2E) and top 15 genera (Figure 2F) with the highest mean relative abundances, which mutually corroborated the significant findings in beta diversity.

### 3.3. Genera Associated with the CAD Spectrum

**Figure 3:**
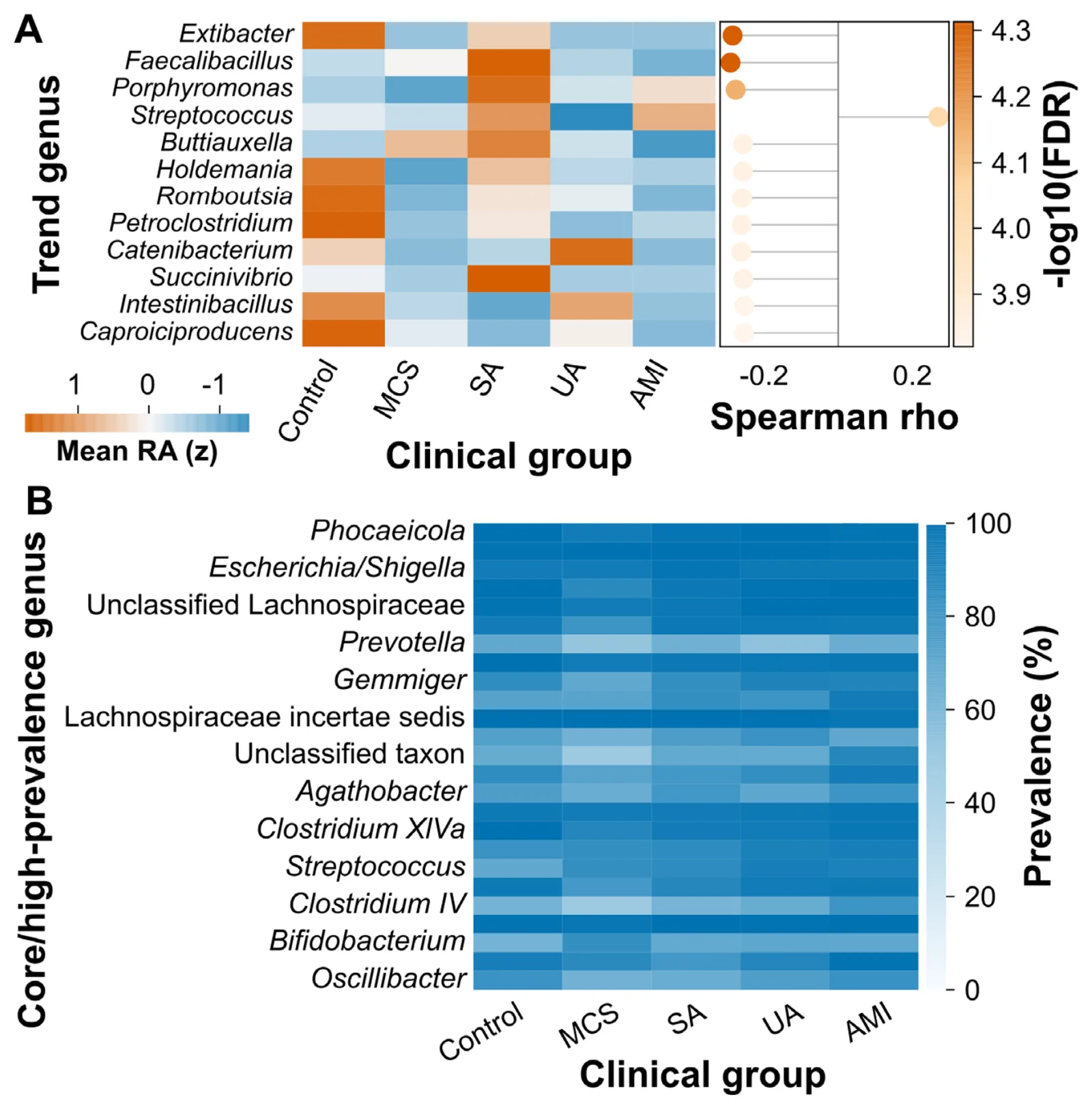
Clinical group-associated genera and cross-group core genera. (**A**) Clinical group-associated genera; the left heatmap displays the Z-scores of the mean relative abundances for the 12 trend genera across the five groups, while the right dot plot shows the Spearman correlation coefficients (ρ) between the CLR-transformed abundances of the corresponding genera and the predefined clinical ordinal ranking, with colors representing the −log_10_^(FDR)^ values. These 12 genera were selected from the top 12 most significant positive and negative correlations ranked by FDR. (**B**) Prevalence (%) of highly prevalent genera across the five groups. Note that this ordinal analysis represents cross-sectional associations and does not imply longitudinal disease progression within individuals.

We subsequently evaluated the correlations between clinical grouping and microbial genera. Using Spearman correlation analysis, we extracted the 12 most significant “trend genera” associated with the predefined group order. Across the clinical spectrum ranging from Non-CAD Control to AMI, a total of 167 genera exhibited significant monotonic associations with the disease progression order (FDR < 0.05). Notably, *Streptococcus* exhibited a positive correlation (ρ = 0.271, FDR = 0.000096; Figure 3A). Its mean relative abundance was relatively low in the Control and MCS groups but was significantly upregulated in the SA and AMI groups, which are characterized by pronounced ischemia or myocardial injury. This suggests that the over-proliferation of Streptococcus and its specific strains may be associated with CAD pathogenesis and severe adverse cardiovascular events, a finding highly consistent with large-scale cardiovascular microbiome studies [14]. The genus *Streptococcus* is generally recognized as a core member of the oral microbiota; its abnormal enrichment in the gut frequently signifies compromised barrier function within the oral–gut axis [14]. Previous studies have demonstrated that specific strains of *Streptococcus* can translocate across the impaired mucosal barrier, enter the systemic circulation, and even colonize localized atherosclerotic plaques [68], potentially contributing to severe inflammatory responses in vascular endothelial cells via their surface virulence factors or peptidoglycan components [25]. The remaining 11 highlighted genera exhibited negative correlations (e.g., *Faecalibacillus*, ρ= −0.291, FDR = 0.000049; Figure 3A). Such correlational patterns across disease categories in this cross-sectional cohort indicate that the gut microecology tends to manifest a depletion of these genera under more severe cardiovascular phenotypes.

Genera such as *Phocaeicola*, *Escherichia/Shigella*, and *Prevotella* demonstrated high prevalences across all five clinical groups (Figure 3B). In our cohort, Streptococcus not only exhibited remarkably high abundance during the AMI stage but also emerged as a core genus with exceptional cross-group prevalence. Under the physiological stress of unstable coronary plaques, the over-proliferation of such opportunistic pathogens may act as an exacerbating factor or exacerbator of acute cardiovascular events [69,70]. Unclassified Lachnospiraceae and *Lachnospiraceae* *incertae sedis* also exhibited high cross-group prevalences. Such high colonization ubiquity suggests that they are likely keystone taxa within the gut microecology of this regional population.

### 3.4. Screening and Ranking of Candidate Markers

**Figure 4:**
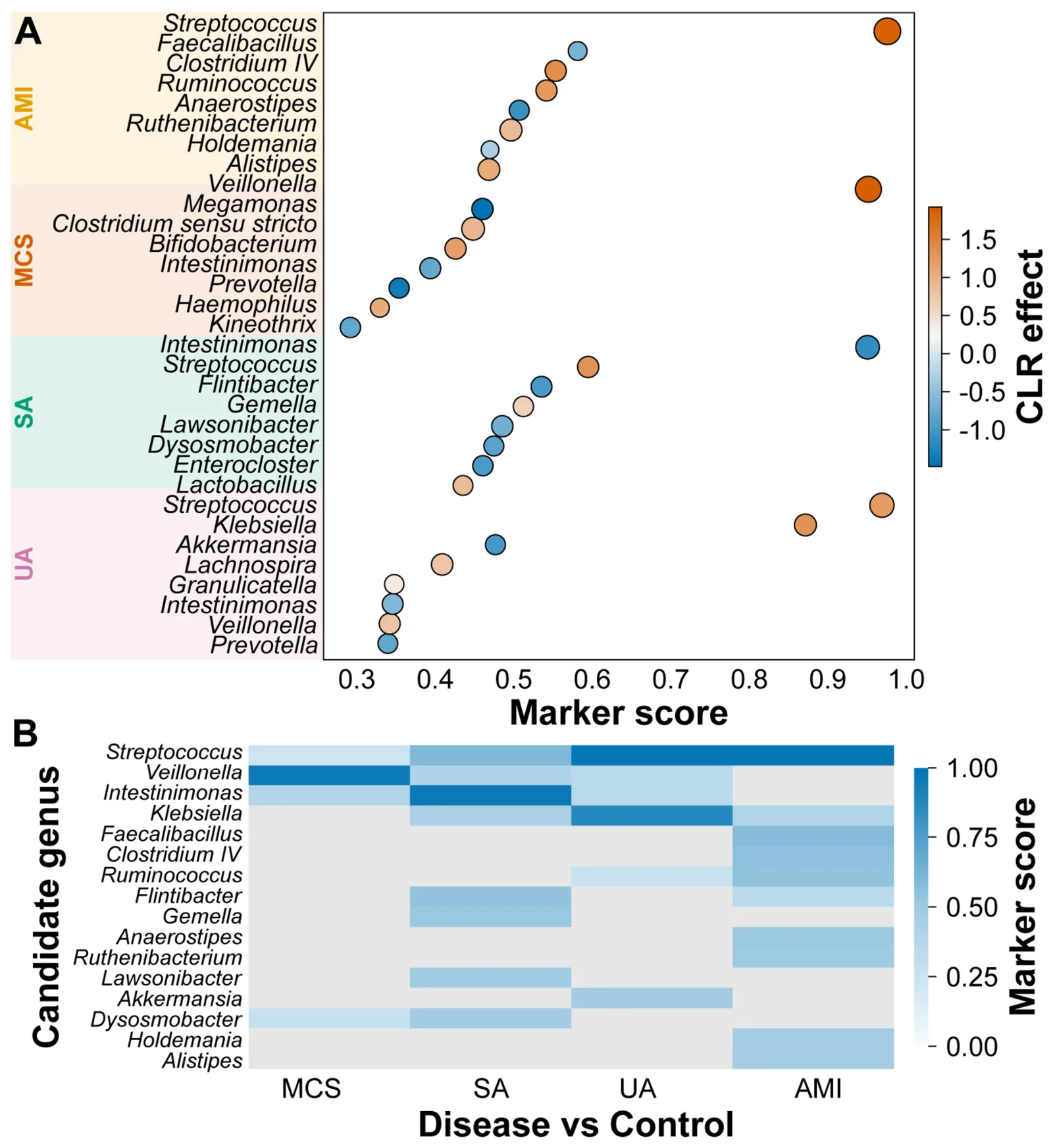
Comprehensive prioritization and cross-subtype distribution of candidate markers across disease subtypes. (**A**) Top 8 genera with the highest Marker scores within each subtype. The *x*-axis represents the Marker score, the dot color indicates the mean CLR difference (direction and magnitude) between the disease group and the Control group, and the dot size reflects the Random Forest feature importance. (**B**) Heatmap of Marker scores for 16 non-redundant candidate genera, selected based on their maximum scores across all subtypes. Gray cells indicate that the genus was not included in the retained candidate set for the corresponding subtype. Note: The Marker score solely indicates candidate priority and does not represent diagnostic probability or external validation performance. A comprehensive 574-row list detailing individual component values and weight sensitivity analyses is provided in Table S3.

In the MCS group, the genus *Veillonella* scored 0.952, with a CLR effect of 1.925 and an FDR of 0.009964 (Figure 4A). *Veillonella* is a genus of Gram-negative anaerobic cocci that typically colonize the oral cavity and intestinal tract [14]. Previous studies have indicated that *Veillonella* may play a critical role in systemic inflammatory responses [71] and the early lipid deposition of arterial plaques [25]. Given that patients in the MCS stage have not yet manifested typical severe ischemic symptoms such as angina [9], the abnormal expansion of intestinal *Veillonella* can be viewed as an early biological warning signal preceding severe structural cardiovascular lesions. The cross-subtype heatmap further revealed that the high scores of this genus were concentrated in the MCS and UA stages, suggesting a specific association with initial plaque formation and its subsequent destabilization (Figure 4B). Progressing to the SA stage, the core indicator microbiota of the disease exhibited a significant shift. *Intestinimonas* emerged as the top-ranked core candidate marker for the SA stage, demonstrating a significantly independent negative correlation (Figure 4A). *Intestinimonas* is a typical butyrate-producing bacterium [72]. As a key metabolite of the gut microbiome, butyrate exerts potent local anti-inflammatory effects and maintains intestinal mucosal barrier integrity by activating G protein-coupled receptors [17]. Highly consistent with the aforementioned analyses, the genus Streptococcus emerged as the core candidate marker in the AMI stage (CLR effect = 1.875, FDR = 0.000338; Figure 4A). The Spearman correlation coefficients between the equal-weight ranking and the original ranking across the MCS, SA, UA, and AMI groups were 0.980, 0.987, 0.984, and 0.863, respectively. In the component-wise leave-one-out sensitivity analysis, the top candidates for MCS, SA, and AMI remained invariant, whereas the top candidate for UA shifted from *Streptococcus* to *Klebsiella* upon the exclusion of the Random Forest importance component (Table S3). The comprehensive weighting components, intra-group rankings, and sensitivity analysis results are detailed in Table S3. Following covariate adjustment, the CLR coefficient for *Intestinimonas* in the SA group was −1.341 (FDR = 0.000998), for Streptococcus in the UA group was 1.374 (FDR = 0.004572), and for Streptococcus in the AMI group was 2.171 (FDR = 0.001213). The covariate-adjusted results and sample sizes for the key candidate markers are summarized in Table 2.

### 3.5. Co-Occurrence Network Topology

Microbial communities form complex co-occurrence networks through intricate interactions, including cooperation, competition, and cross-feeding. The topological evolution of these networks serves as a crucial dimension for evaluating ecosystem stability and disease progression [73]. Utilizing a candidate set of 40 shared genera—filtered based on global prevalence and mean relative abundance—we estimated the conditional dependence networks for the five original clinical groups using the SPIEC-EASI MB method coupled with StARS model selection. This approach allowed us to analyze the evolutionary trajectory of intra-network interactions among core genera across distinct CAD stages.

**Figure 5:**
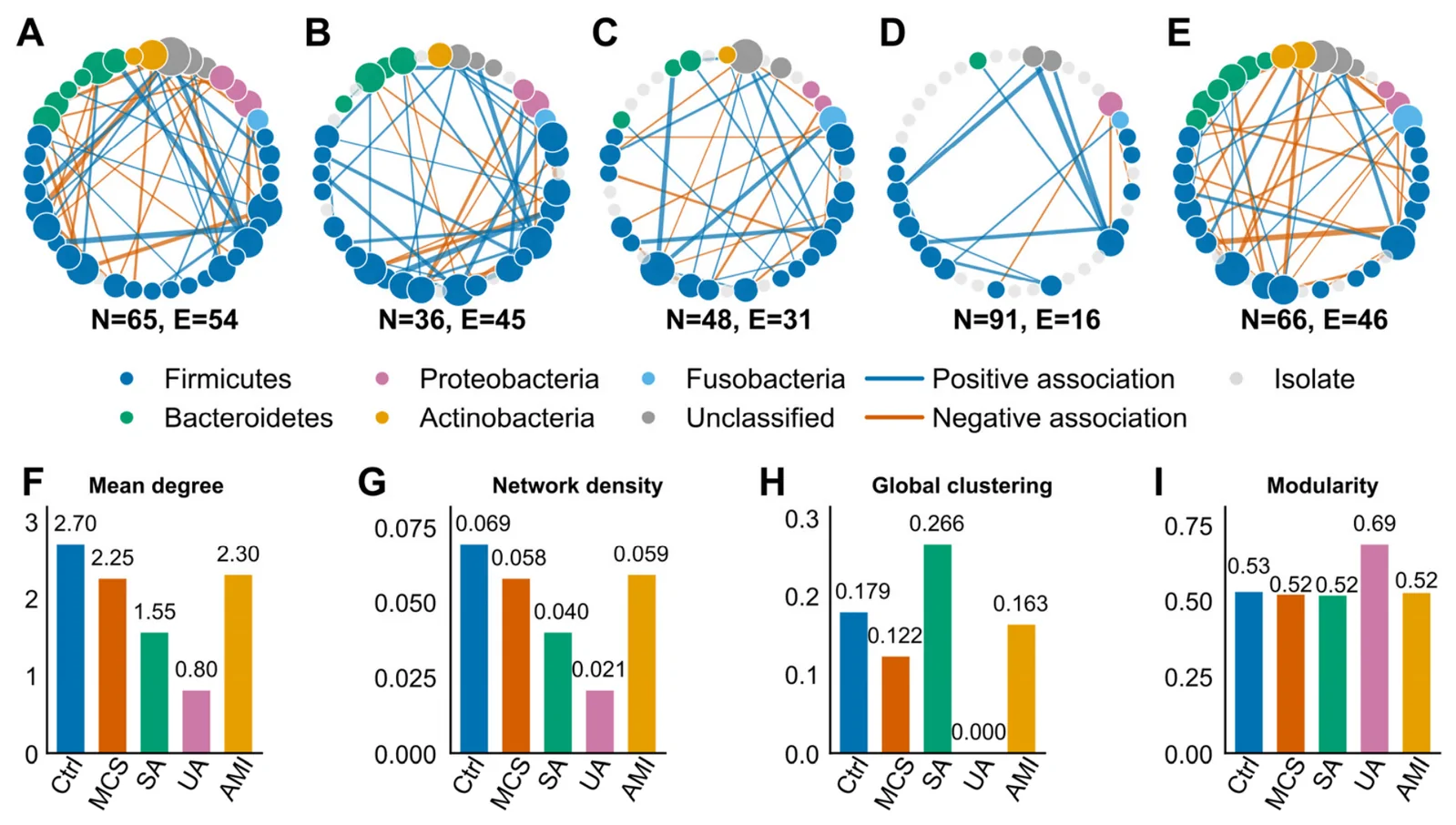
SPIEC-EASI conditional dependence networks and topological summaries across the five clinical groups. (**A**–**E**) Networks for the Control, MCS, SA, UA, and AMI groups, respectively. All five groups utilized an identical candidate set of 40 genera and fixed node coordinates (arranged in a circular layout based on phylum and mean abundance). Node color indicates the phylum-level classification, while node size is proportional to its degree; gray nodes represent isolated nodes. Blue and orange edges denote positive and negative conditional dependencies, respectively, with edge width proportional to the absolute magnitude of the conditional association. N and E in each panel denote the sample size and the number of retained edges, respectively. (**F**–**I**) Mean degree, network density, global clustering coefficient, and Louvain modularity. Gray cells indicate that the genus was not included in the retained candidate set for the corresponding subtype. Note: Given that only a single network was fitted per group and sample sizes varied across groups, the observed topological differences were not subjected to statistical inferential testing and are intended solely for descriptive comparisons.

As the disease progressed from the Control to the UA stage, the total number of edges exhibited a continuous decline from 54 in the Control group to 16 in the UA group (Figure 5A–D). Concurrently, the mean degree dropped from 2.70 to 0.80 (Figure 5F), and the network density decreased from 0.069 to 0.021 (Figure 5G). Throughout this progression, there was a significant increase in isolated nodes that lost effective associations with other microbial taxa within the network (Figure 5A–D). Such a severe depletion of ecological connectivity during the UA stage signifies that the gut microecological barrier function had reached a highly vulnerable critical threshold. However, upon progression to the AMI stage, the number of retained nodes recovered to 34, and the total number of edges rebounded to 46, with both the mean degree and network density returning to levels comparable to those observed in the MCS group. The number of connected components also reconverged to 8 (Figure 5E). Notably, while positive edges consistently outnumbered negative edges in the first four groups, the AMI network exhibited a reversed pattern, where the number of negative edges (n = 28) surpassed that of positive edges (n = 18).

It should be noted that network edges represent conditional dependencies rather than direct microbial interactions. Furthermore, given the unequal sample sizes across groups, the observed topological differences are intended solely for descriptive comparisons. A comprehensive summary of the topological metrics is provided in Table S4.

### 3.6. Internal Model Performance, Clinical Relevance, and Covariate Adjustment

**Figure 6:**
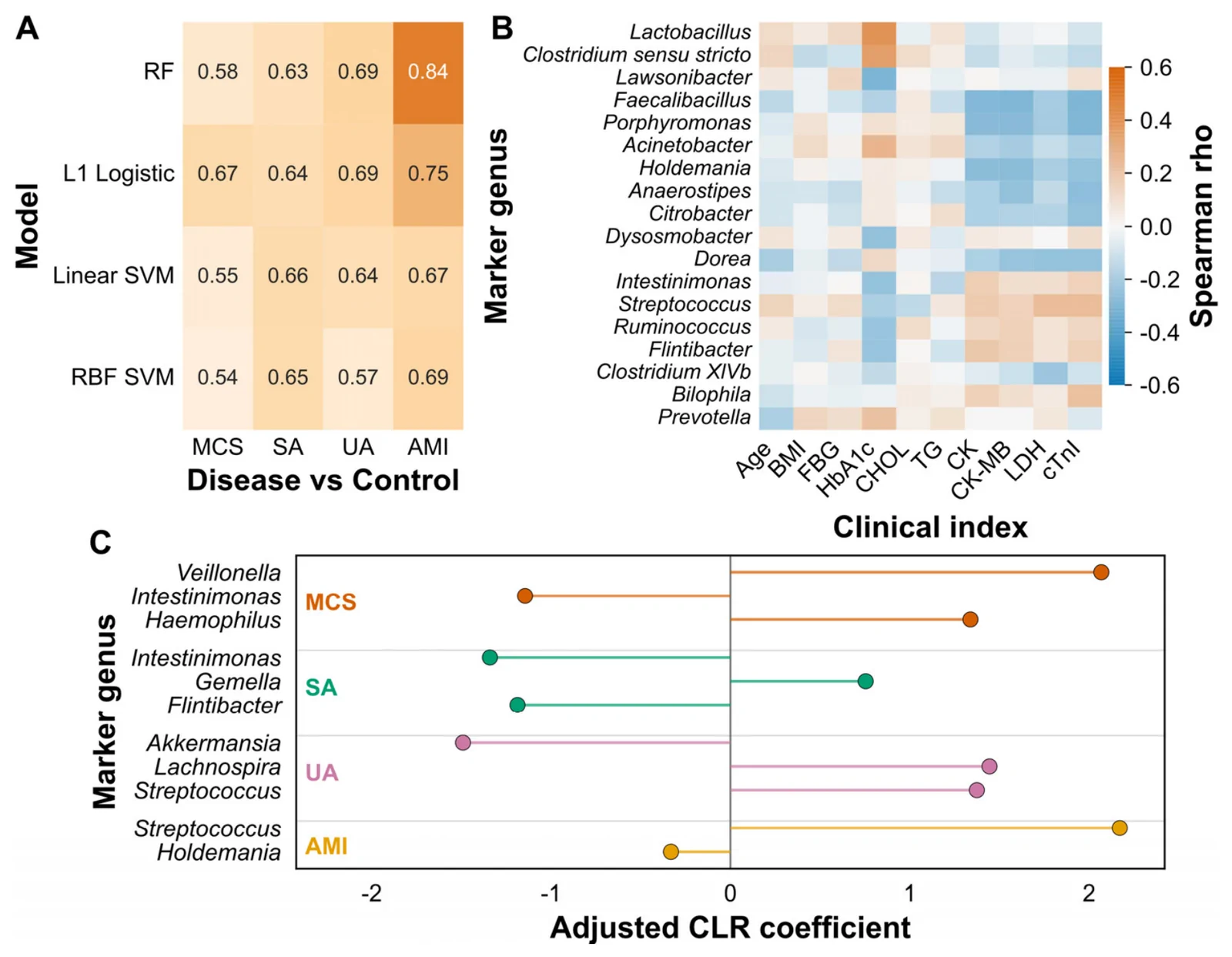
Internal classification model performance, candidate marker-clinical correlations, and covariate adjustment results. (**A**) Mean AUCs of random forest (RF), L1-regularized logistic regression, linear support vector machine (SVM), and radial basis function (RBF) kernel SVM in comparisons between each subtype and Control; each cell represents the mean of 15 validation folds obtained from 3 repeats of 5-fold repeated stratified cross-validation. The total sample sizes for the MCS, SA, UA, and AMI tasks were 101, 113, 156, and 131, with input genera counts of 133, 144, 148, and 149, respectively; results represent internal validation performance only. (**B**) Spearman correlation matrix between 18 candidate genera (the union of the top 15 genera by marker score for each subtype, selected based on the maximum absolute Spearman’s ρ across clinical indicators) and 10 clinical indicators; cell values and colors both represent ρ, with a minimum effective sample size of 20 for each combination. Complete FDR-significant results are presented in Table S5. (**C**) Up to 3 adjusted associations with FDR < 0.05 shown per subtype; points represent adjusted centered log-ratio (CLR) regression coefficients for disease status, and horizontal line segments connect to zero. Models were adjusted for age, BMI, sex, hypertension, type 2 diabetes mellitus, and hypertriglyceridemia. The complete-case analysis sample sizes for MCS, SA, UA, and AMI were 100, 112, 155, and 115, respectively; complete results are provided in Table S6.

To evaluate the classification and predictive capabilities, we conducted internal cross-validation using four mainstream machine learning algorithms. In the MCS stage, the mean area under the curve (AUC) across all models was generally low (0.54–0.67), with L1-regularized Logistic Regression yielding the best performance (AUC = 0.666; Figure 6A). L1 regularization is renowned for its robust feature-sparsifying capability [44]. This suggests that during the very early, subclinical ischemic state of CAD, the dysbiotic signals within the microecology are relatively weak and linear, necessitating stringent feature filtration for capture. Conversely, in the AMI stage, model performance exhibited a substantial leap (AUC ranging from 0.67 to 0.84), with the non-linear Random Forest (RF) algorithm achieving an AUC of 0.844 ± 0.067 (Figure 6A). As a non-linear algorithm based on decision tree ensembles, RF excels at capturing complex interactive features within high-dimensional data [74]. This indicates that during the AMI stage, the overall structure of the gut microbiota is not only disrupted [75] but also harbors highly specific non-linear features sufficient to discriminate these patients from the non-CAD population. We subsequently constructed a Spearman correlation matrix to directly associate the 18 candidate core genera with 10 critical clinical indices (Figure 6B). Notably, *Faecalibacillus* exhibited a negative correlation with cTnI (ρ = −0.292, FDR = 0.000185), suggesting that the depletion of certain commensal bacteria in the gut may compromise the host’s capacity to withstand acute physiological stress. *Streptococcus* demonstrated a positive correlation with cTnI (ρ = 0.226, FDR = 0.004168), as well as varying degrees of positive correlations with CK, CK-MB, and LDH. Furthermore, the correlation blocks for the majority of candidate genera with routine metabolic indices such as BMI and FBG were relatively pale (weak correlations). This indicates that the selected markers are not merely by-products of obesity or hyperglycemia. Finally, we employed multiple regression models to examine the candidate markers while adjusting for age, BMI, sex, hypertension, type 2 diabetes mellitus, and hypertriglyceridemia. Following the removal of confounding effects from these six common clinical factors, the core markers for each subtype maintained exceptionally robust significance (FDR < 0.05). In the MCS group, the adjusted coefficient for *Veillonella* was 2.068 (FDR = 0.002603), confirming that its enrichment is not an artifact of comorbid conditions like hypertension in early-stage patients, but rather an independent marker of early disease. In the UA group, the adjusted coefficient for *Akkermansia* was −1.491 (FDR = 0.001196), while the adjusted CLR coefficient for Streptococcus remained above 2.0. The significant FDR-corrected correlations and the comprehensive results of the adjusted models are provided in Table S5 and Table S6, respectively.

## 4. Discussion

Through the application of stringent clinical diagnostic criteria, we successfully established a high-quality clinical cohort comprising 306 individuals, encompassing various progression stages of coronary artery disease (CAD)—namely MCS, SA, UA, and AMI—alongside a non-CAD Control group. The distribution patterns of the baseline characteristics not only highly align with the clinicopathological evolutionary traits of CAD but also effectively eliminate a substantial amount of potential confounding interference for subsequent differential analyses of the gut microecology [28]. A striking finding within the baseline analysis is the absence of statistically significant differences across the five clinical groups regarding traditional metabolic risk factors, including BMI, FBG, CHOL, and TG. This phenomenon suggests that underlying metabolic abnormalities may be ubiquitously present among this specific clinical population presenting with cardiovascular symptoms [76] (or alternatively, this uniformity may result from the homogenizing effects of long-term statin or hypoglycemic therapies [77,78]). More importantly, it reflects an emerging consensus in contemporary atherosclerosis research: the clinical deterioration from SA to AMI is typically not determined by simple elevations in basal lipid levels, but is rather driven by plaque vulnerability and drastic alterations in the localized endothelial microenvironment [7,79]. This observation serendipitously validates the rationale for introducing the gut microbiome as a variable in this study, implying that specific dysbiosis of the gut microbiota may be intrinsically associated with CAD pathogenesis.

Historically, many early microbiome studies have treated coronary artery disease (CAD) as a single binary variable, a practice prone to masking true biological signals owing to the substantial internal heterogeneity of the disease [14,80]. Our results demonstrated that when all CAD patients were pooled into a single group and compared with the control group, neither the alpha diversity nor the beta diversity of the gut microbiota exhibited statistically significant differences. In fact, atherosclerosis is a protracted and continuous process spanning from lipid deposition and plaque formation to plaque rupture [11,81,82]. By finely partitioning CAD into four distinct stages for a five-group joint analysis, this study demonstrates the necessity of refined clinical phenotypic staging for cardiovascular cohorts in microbiome research.

By spanning the complete cross-sectional clinical spectrum from Control to AMI, we successfully characterized the dynamic trajectory of the gut microecology accompanying the progression of ischemic severity. Within this cohort, *Streptococcus* not only exhibited remarkably high abundance in the AMI stage but also emerged as a core genus with a high detection rate across all groups. In sharp contrast to the expansion of *Streptococcus*, 11 trending genera, including *Faecalibacillus*, demonstrated a significant negative correlation along the disease deterioration gradient. This reflects a tendency of the gut microecology to exhibit a depletion of these commensal bacteria under more severe cardiovascular phenotypes [75].

Taxa belonging to the phylum Firmicutes, such as *Faecalibacillus*, typically participate in complex carbohydrate fermentation in the gut of healthy individuals, producing core metabolites such as short-chain fatty acids (SCFAs) that possess potent anti-inflammatory properties and maintain the integrity of the intestinal mucosal barrier [17,83]. The depletion of beneficial bacteria during the UA and AMI stages may lead to increased intestinal permeability, leaving the circulatory system chronically exposed to pro-inflammatory substances such as endotoxins, which in turn indirectly drives the exacerbation of coronary lesions [20,84].

However, 16S rRNA sequencing can only provide abundance association information at the genus or family level, making it difficult to distinguish specific strains within the same genus that possess vastly different metabolic functions [85]. Faced with these core markers exhibiting strong correlations across clinical cross-sections, relying solely on broad-scale analyses at the metagenomic or amplicon level is no longer sufficient to unravel the underlying causal mechanisms [86]. Future research urgently needs to advance to the single-strain level by utilizing targeted isolation and culturomics approaches to precisely isolate these frequently occurring single strains of Lachnospiraceae or *Streptococcus* from patient feces [86]. Upon obtaining pure cultures, conducting single-strain whole-genome sequencing analysis alongside gnotobiotic animal mono-colonization experiments is essential to robustly elucidate the precise functions and causal pathways of these core strains within the host.

Relying solely on statistical *p*-values is often insufficient to bridge the gap from association to clinical diagnostic biomarker [87,88]. We innovatively introduced a Marker Score evaluation system that integrates multidimensional parameters. This system not only successfully identified candidate genera with high diagnostic potential but, more importantly, revealed that CAD does not correspond to a single, static dysbiosis pattern; rather, it exhibits a dynamically alternating evolutionary trajectory accompanying the degree of ischemia. Analysis results indicated that *Veillonella* serves as a specific indicator bacterium during the MCS stage, the very early phase of CAD. Crucially, in multivariate regression models, even after strictly adjusting for numerous confounding factors such as age, BMI, hypertension, and diabetes, this association remained highly significant, highlighting its clinical value. *Intestinimonas* emerged as the top-ranking core candidate biomarker for the SA stage; its specific and profound depletion during this stage reflects a severe impairment of the host’s intestinal anti-inflammatory and metabolic protection mechanisms, which may provide a microenvironment promoting plaque progression toward more severe ischemic states [14,89]. *Streptococcus* became the core candidate biomarker in both the UA and AMI stages; this trajectory—spanning almost the entire middle-to-late phases of ischemia and ultimately erupting in the acute phase—profoundly underscores its independent pathological value in acute ischemic events. By contrast, although genera such as *Faecalibacillus* and *Anaerostipes* ranked high in Marker Score during initial screening, they lost statistical significance following multivariate adjustment. This suggests that microecological dysbiosis during the AMI stage may be driven by the outbreak of opportunistic pathogens represented by *Streptococcus*, rather than merely the depletion of commensal bacteria [68,75]. This enrichment of pathogenic bacteria, independent of patients’ baseline physiological indices, further supports the hypothesis that “microbial translocation and local plaque infection” are associated with AMI onset [68].

In gut microecological research, abundance variations in a single microbial taxon are often insufficient to fully reflect the overall health status of the ecosystem [90,91]. Our results demonstrated that during disease progression from Control to MCS, SA, and even UA, the gut microbiota network exhibited continuous deconstruction and fragmentation. In microecological theory, highly interconnected networks typically imply stronger ecological adaptability and anti-interference capability [92]. The dense network topology of the Control group reflects widespread metabolic cooperation and homeostatic constraints within the healthy gut [92]. Notably, although the overall network connectivity in the SA stage decreased compared with the Control group, its global clustering coefficient reached the highest value among the five groups. This phenomenon suggests that under prolonged chronic ischemia and metabolic stress, certain key taxa in the SA stage establish highly dense local cooperative networks, forming a local compensatory mechanism within the harsh intestinal microenvironment [93,94]; this may also explain why the clinical condition of SA patients can remain relatively stable over extended periods. The most clinically illuminating finding from the network analysis lies in the anomalous rebound and rewiring of the network during the AMI stage, which may represent a severe pathological stress response. From a pathophysiological perspective, the cardiogenic shock, acute intestinal hypoperfusion, and drastic sympathetic nervous activation accompanying AMI onset induce catastrophic alterations in the intestinal internal environment [95,96]. This antagonism-dominated network topological feature not only corroborates the sharp decline in species diversity observed in the AMI group but also explains the microecological shifts under acute cardiovascular events. It should be noted that, due to the application of single-network fitting and differing sample sizes across groups, topological characteristics between groups are intended solely for descriptive comparisons. Nevertheless, the consistent ecological evolutionary patterns described above provide a novel theoretical basis for future multi-omics network intervention strategies, such as targeting specific hub node strain combinations to delay CAD progression.

During the translation into clinical diagnostic tools, the generalization capability of models, the mechanistic link between markers and host phenotypes, and the independence from clinical confounding factors constitute the core tests [97,98]. Our clinical correlation matrix successfully bridged the gut microbiota with the host’s myocardial pathological phenotypes. Cardiac troponin I (cTnI) and creatine kinase-MB (CK-MB) are clinically recognized gold standards for myocardial ischemic necrosis [28]. Correlation analysis revealed that commensal bacteria such as *Faecalibacillus* were significantly negatively correlated with cTnI (FDR < 0.001), whereas Streptococcus showed significant positive correlations with cTnI, CK, and LDH. This simultaneous manifestation of protective bacteria depletion and pathogenic bacteria expansion within the same individual profoundly elucidates the role of intestinal barrier impairment in acute cardiovascular events. More importantly, the associations between the majority of candidate genera and routine metabolic indices such as BMI and FBG were exceptionally weak. Over the past decade, numerous cardiovascular microbiome studies have frequently been hampered by the confounding effects of obesity and diabetes backgrounds [99]. Our results robustly demonstrate that the identified microecological biomarkers reflect CAD-specific ischemic and vascular lesion characteristics rather than being mere by-products of metabolic syndrome. Following the adjustment for confounding factors, the core markers of each subtype maintained exceptionally high robustness. The adjusted coefficient for *Veillonella* in the MCS group remained as high as 2.068, proving that its enrichment is independent of common early comorbidities such as hypertension, making it a genuine independent biomarker for early vascular lesions. *Akkermansia* in the UA group exhibited a significant and independent negative correlation after adjustment. *Akkermansia* is a widely recognized next-generation probiotic (NGP), which can degrade intestinal mucins, maintain the physical thickness of the mucosal barrier, and suppress systemic inflammation [100]. Its specific and independent depletion during the UA stage carries profound clinical significance, strongly suggesting that the loss of intestinal barrier protective mechanisms is closely associated with the transition from stable plaques to vulnerable plaques. The adjusted coefficient for *Streptococcus* in the AMI stage still exceeded 2.0, demonstrating its potential as an AMI-specific independent microecological biomarker.

Finally, this study has several limitations. Importantly, detailed records of drug therapies (such as statins, beta-blockers, antibiotics, and probiotics) and lifestyle factors (including diet, smoking, alcohol consumption, and physical activity) were not fully available in our retrospective dataset. These factors are known to significantly influence gut microbiota composition and represent potential sources of unmeasured confounding [101,102,103]. Future prospective studies with comprehensive clinical and lifestyle metadata are required to isolate the independent microecological signals of CAD progression.

In summary, through internal machine learning validation and rigorous clinical covariate adjustment, this study effectively eliminated false positives and demonstrated the tremendous clinical translational potential of gut microbiota signatures as precise staging and non-invasive auxiliary diagnostic tools for CAD.

## Figures and Tables

**Table 1:** Clinical characteristics of the five study groups.

Characteristic	Control (N = 65)	MCS (N = 36)	SA (N = 48)	UA (N = 91)	AMI (N = 66)	Test Statistic	*p*-Value	FDR-Adjusted *p*
Age (years)	53.00 [44.00, 63.00] (n = 65)	64.00 [57.50, 68.25] (n = 36)	58.50 [50.75, 65.00] (n = 48)	63.00 [55.00, 70.00] (n = 91)	55.00 [46.00, 65.75] (n = 66)	H = 29.14	<0.001	<0.001
Body mass index (kg/m^2^)	24.10 [21.89, 26.19] (n = 64)	24.08 [22.52, 27.38] (n = 36)	24.16 [22.05, 25.80] (n = 48)	24.97 [22.49, 27.22] (n = 91)	24.80 [22.57, 26.58] (n = 51)	H = 4.26	0.372	0.397
FBG (mmol/L)	5.15 [4.89, 6.05] (n = 65)	5.49 [4.93, 5.95] (n = 36)	5.27 [4.88, 5.85] (n = 46)	5.45 [5.00, 6.50] (n = 90)	5.64 [4.93, 6.56] (n = 65)	H = 6.85	0.144	0.179
HbA1c (%)	-- (n = 0)	-- (n = 0)	6.52 [6.12, 6.92] (n = 2)	6.90 [6.63, 9.28] (n = 8)	5.69 [5.56, 7.18] (n = 26)	H = 5.81	0.055	0.087
Total cholesterol (mmol/L)	4.12 [3.69, 4.88] (n = 58)	3.98 [3.34, 4.95] (n = 35)	4.37 [3.72, 5.00] (n = 46)	3.96 [3.36, 5.10] (n = 86)	3.99 [3.45, 4.71] (n = 42)	H = 4.06	0.398	0.398
Triglycerides (mmol/L)	1.30 [1.05, 1.97] (n = 61)	1.44 [1.09, 1.92] (n = 35)	1.65 [1.20, 2.00] (n = 46)	1.61 [1.18, 2.25] (n = 87)	1.63 [1.26, 1.96] (n = 41)	H = 6.83	0.145	0.179
CK (U/L)	69.00 [59.00, 83.00] (n = 53)	77.00 [50.00, 104.00] (n = 29)	84.00 [59.00, 105.00] (n = 42)	87.00 [59.25, 116.00] (n = 86)	264.00 [113.00, 740.00] (n = 61)	H = 67.99	<0.001	<0.001
CK-MB (U/L)	0.90 [0.70, 1.40] (n = 53)	0.90 [0.70, 1.50] (n = 29)	1.25 [0.80, 1.98] (n = 42)	1.25 [0.90, 2.08] (n = 86)	11.60 [1.90, 49.48] (n = 62)	H = 83.54	<0.001	<0.001
LDH (U/L)	151.50 [138.00, 168.75] (n = 54)	161.00 [145.00, 175.00] (n = 29)	164.50 [148.00, 188.75] (n = 42)	166.00 [148.25, 189.75] (n = 86)	359.00 [195.00, 736.00] (n = 57)	H = 79.36	<0.001	<0.001
cTnI (ng/mL)	0.00 [0.00, 0.00] (n = 53)	0.00 [0.00, 0.00] (n = 30)	0.00 [0.00, 0.00] (n = 41)	0.00 [0.00, 0.00] (n = 87)	4.00 [0.15, 22.92] (n = 62)	H = 153.10	<0.001	<0.001
Male sex, n (%)	24 (36.9%)	21 (58.3%)	27 (56.2%)	54 (59.3%)	61 (92.4%)	chi-square = 43.95	<0.001	<0.001
Hypertension, n (%)	25 (38.5%)	29 (80.6%)	20 (41.7%)	60 (65.9%)	31 (47.0%)	chi-square = 26.00	<0.001	<0.001
Type 2 diabetes, n (%)	8 (12.3%)	6 (16.7%)	11 (22.9%)	23 (25.3%)	12 (18.2%)	chi-square = 4.67	0.323	0.369
Hypertriglyceridemia, n (%)	7 (10.8%)	0 (0.0%)	2 (4.2%)	2 (2.2%)	4 (6.1%)	chi-square = 8.33	0.080	0.117
Cardiac neurosis, n (%)	17 (26.2%)	0 (0.0%)	0 (0.0%)	0 (0.0%)	0 (0.0%)	chi-square = 66.74	<0.001	<0.001
Coronary artery muscle bridge, n (%)	22 (33.8%)	9 (25.0%)	11 (22.9%)	1 (1.1%)	1 (1.5%)	chi-square = 48.06	<0.001	<0.001

Continuous variables are median [IQR] with group-specific non-missing n; categorical variables are n (%). H denotes the Kruskal–Wallis statistic and chi-square denotes Pearson’s chi-square statistic. *p* values were adjusted across baseline characteristics by the Benjamini–Hochberg procedure. Laboratory measurement units were not supplied in the source metadata and require author verification before submission.

**Table 2:** Covariate-adjusted associations for the top five candidate genera in each disease subtype.

Subtype	Selection Rank	Genus	Adjusted CLR Coefficient	FDR-Adjusted *p*	n
AMI	1	*Streptococcus*	2.171	0.001	115
AMI	2	*Faecalibacillus*	−0.440	0.104	115
AMI	3	*Clostridium IV*	0.849	0.116	115
AMI	4	*Ruminococcus*	0.797	0.212	115
AMI	5	*Anaerostipes*	−0.921	0.065	115
MCS	1	*Veillonella*	2.068	0.003	100
MCS	2	*Megamonas*	−0.441	0.562	100
MCS	3	*Clostridium sensu stricto*	0.966	0.082	100
MCS	4	*Bifidobacterium*	1.043	0.097	100
MCS	5	*Intestinimonas*	−1.145	0.018	100
SA	1	*Intestinimonas*	−1.341	<0.001	112
SA	2	*Streptococcus*	1.439	0.011	112
SA	3	*Flintibacter*	−1.187	0.008	112
SA	4	*Gemella*	0.754	0.003	112
SA	5	*Lawsonibacter*	−0.707	0.011	112
UA	1	*Streptococcus*	1.374	0.005	155
UA	2	*Klebsiella*	1.271	0.018	155
UA	3	*Akkermansia*	−1.491	0.001	155
UA	4	*Lachnospira*	1.444	0.004	155
UA	5	*Granulicatella*	0.478	0.042	155

Candidate genera were ranked by marker score in Figure 4 (top five per subtype). Adjusted CLR coefficients are from subtype-versus-Control ordinary least-squares models adjusted for age, body mass index, sex, hypertension, type 2 diabetes, and hypertriglyceridemia. FDR-adjusted *p*-values are across 60 models; n denotes the analysis sample size.

## Data Availability

The original data presented in the study are openly available in [NCBI Sequence Read Archive (SRA) database] at [BioProject ID PRJNA779598].

## Supplementary Materials

The following supporting information can be downloaded at https://www.mdpi.com/article/10.3390/microorganisms14092117/s1, Figure S1: Sample quality control and OTU filtering; Figure S2: Overall comparison between the Control and CAD groups; Table S1: Sample-level sequencing quality-control information; Table S2: Alpha-diversity and beta-diversity test statistics; Table S3: Complete subtype-specific marker-score components, ranking, and sensitivity analyses; Table S4: SPIEC-EASI topology summary for the five clinical groups; Table S5: FDR-significant marker-clinical correlations; Table S6: Covariate-adjusted subtype-versus-Control candidate-marker models.
