# Supplementary material for: Clinical Subtype Stratification Reveals Gut Microbial Alterations, Candidate Markers, and Co-Occurrence Network Remodeling in Coronary Artery Disease
Source: Microorganisms. 2026 Sep 21;14(9):2117. doi: 10.3390/microorganisms14092117 (PMC13609194; doi:10.3390/microorganisms14092117)
Supplement: Supplementary file 1 [file microorganisms-14-02117-s001.zip › Supplementary figures/Figure S1. Sample quality control and OTU filtering.pdf]

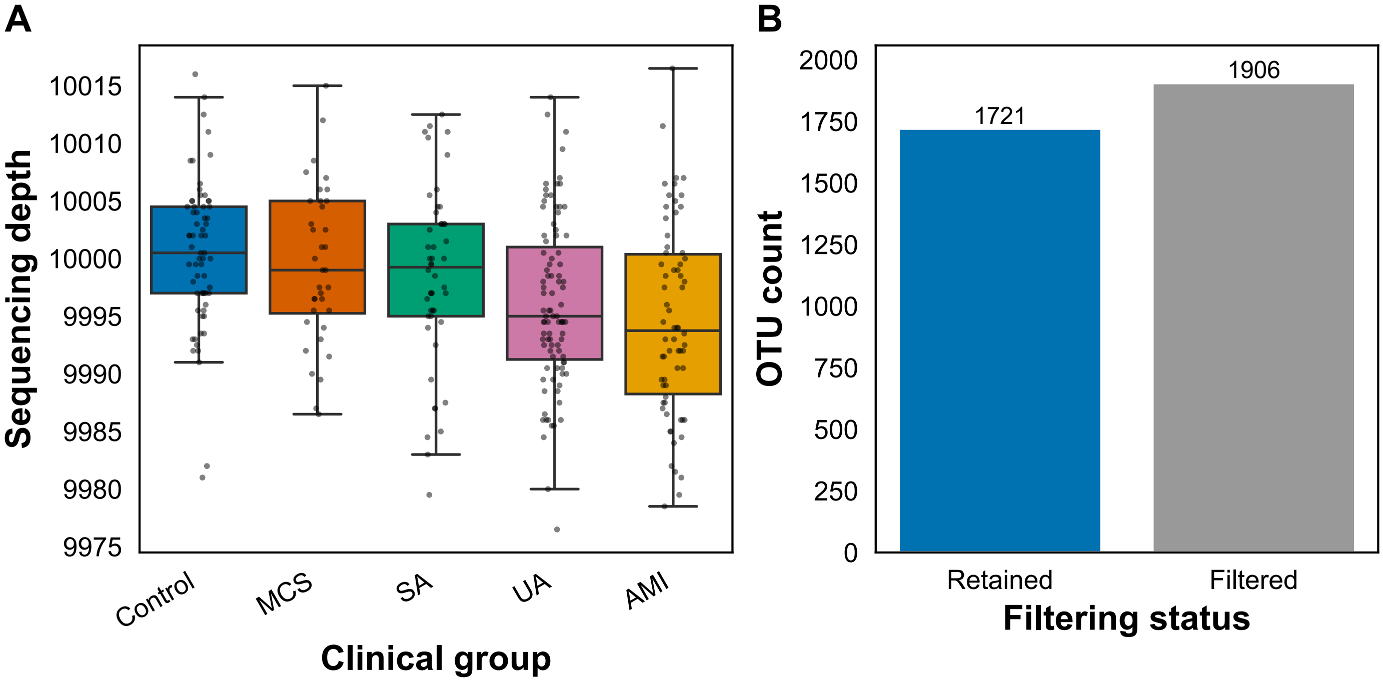
A
Sequencing depth
10015
10010
10005
10000
9995
9990
9985
9980
9975
Control
MCS
SA
UA
AMI
Clinical group
B
OTU count
2000
1750
1500
1250
1000
750
500
250
0
1721
1906
Retained
Filtered
Filtering status
