# Supplementary material for: Clinical Subtype Stratification Reveals Gut Microbial Alterations, Candidate Markers, and Co-Occurrence Network Remodeling in Coronary Artery Disease
Source: Microorganisms. 2026 Sep 21;14(9):2117. doi: 10.3390/microorganisms14092117 (PMC13609194; doi:10.3390/microorganisms14092117)
Supplement: Supplementary file 1 [file microorganisms-14-02117-s001.zip › Supplementary figures/Figure S2. Overall comparison between the Control and CAD groups.pdf]

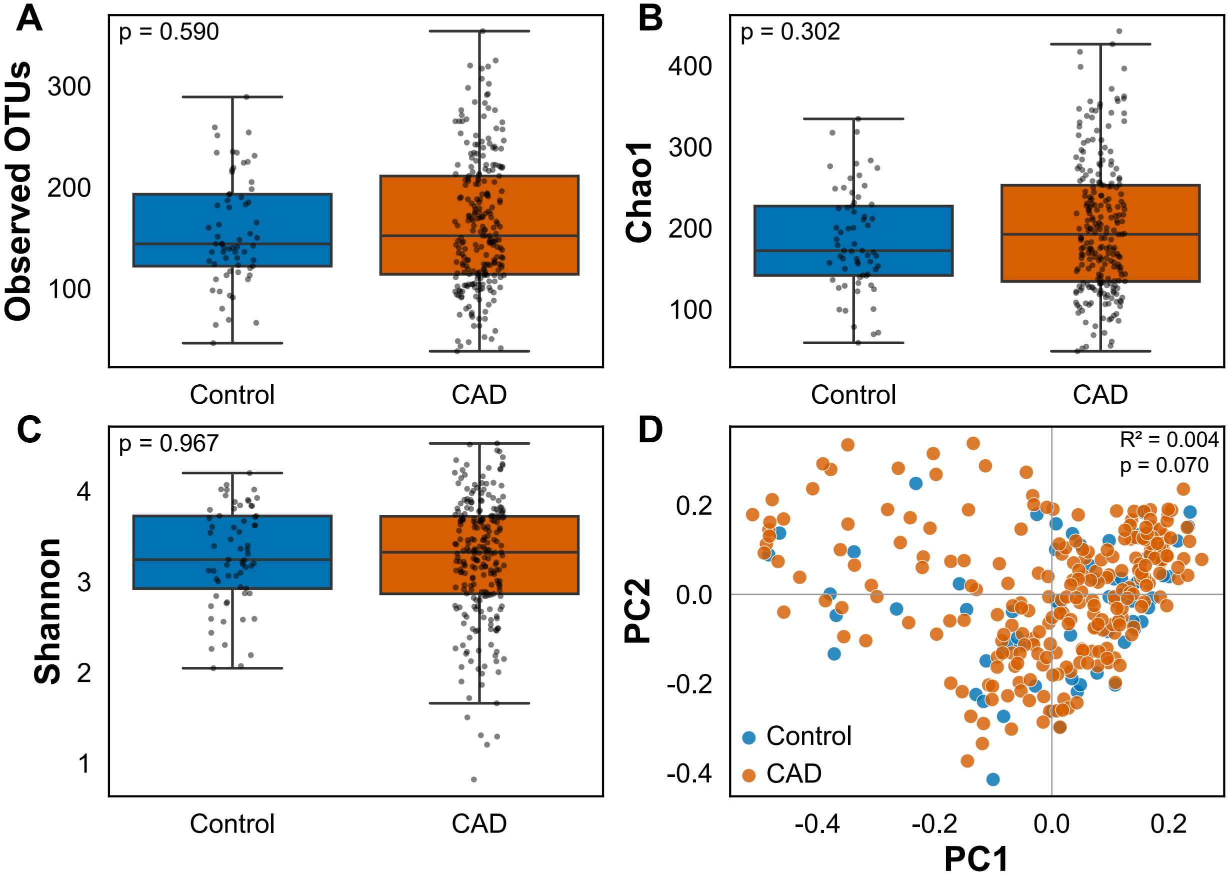
A
p = 0.590
Observed OTUs
300
200
100
Control
CAD
B
p = 0.302
Chao1
400
300
200
100
Control
CAD
C
p = 0.967
Shannon
4
3
2
1
Control
CAD
D
R² = 0.004
p = 0.070
PC2
0.2
0.0
-0.2
-0.4
Control
CAD
-0.4
-0.2
0.0
0.2
PC1
